# Diurnal Expression Pattern, Allelic Variation, and Association Analysis Reveal Functional Features of the *E1* Gene in Control of Photoperiodic Flowering in Soybean

**DOI:** 10.1371/journal.pone.0135909

**Published:** 2015-08-14

**Authors:** Hong Zhai, Shixiang Lü, Hongyan Wu, Yupeng Zhang, Xingzheng Zhang, Jiayin Yang, Yaying Wang, Guang Yang, Hongmei Qiu, Tingting Cui, Zhengjun Xia

**Affiliations:** 1 Key Laboratory of Soybean Molecular Design Breeding, Northeast Institute of Geography and Agroecology, Chinese Academy of Sciences, Harbin, China; 2 University of Chinese Academy of Sciences, No.19A Yuquan Road, Beijing, China; 3 Huaiyin Institute of Agricultural Sciences of Xuhuai Region in Jiangsu, Huaian, China; 4 Soybean Research Institute, Jilin Academy of Agricultural Sciences, Changchun, China; University of Missouri, UNITED STATES

## Abstract

Although four maturity genes, *E1* to *E4*, in soybean have been successfully cloned, their functional mechanisms and the regulatory network of photoperiodic flowering remain to be elucidated. In this study, we investigated how the diurnal expression pattern of the *E1* gene is related to photoperiodic length; and to what extent allelic variation in the B3-like domain of the *E1* gene is associated with flowering time phenotype. The bimodal expression of the *E1* gene peaked first at around 2 hours after dawn in long-day condition. The basal expression level of *E1* was enhanced by the long light phase, and decreased by duration of dark. We identified a 5bp (3 SNP and 2-bp deletion) mutation, referred to an *e1-b3a*, which occurs in the middle of B3 domain of the *E1* gene in the early flowering cultivar Yanhuang 3. Subcellular localization analysis showed that the putative truncated e1-b3a protein was predominately distributed in nuclei, indicating the distribution pattern of e1-b3a was similar to that of E1, but not to that of e1-as. Furthermore, genetic analysis demonstrated allelic variations at the *E1* locus significantly underlay flowering time in three F_2_ populations. Taken together, we can conclude the legume specific *E1* gene confers some special features in photoperiodic control of flowering in soybean. Further characterization of the *E1* gene will extend our understanding of the soybean flowering pathway in soybean.

## Introduction

Soybean provides human beings with both good quality protein and oil. As early as the 1920s, researchers used soybean and other crop species as a model to study flowering time photoperiodic response, leading to the discovery and the advancement of photoperiodism [1–3]. In soybean, about ten genes (*E1* to *E9*, and *J*) controlling the flowering time have been genetically mapped or identified [4–12]. Of them, four *E* genes, *E1*, *E2*, *E3* and *E4* have been successfully cloned [13–16]. In general, *GIGANTEA* (*GI*) promotes flowering in long day (LD) plants and inhibits flowering in short day (SD) plants [17]. *GI* functions in circadian period determination, light inhibition of hypocotyl elongation, and responses to multiple abiotic stresses in *Arabidopsis* as well as in *Brassica rapa* [18]. Natural variation in the *GI* gene is responsible for a major quantitative trait locus in circadian period in *Brassica rapa* [18]. In soybean, positional cloning identified that the causal gene for the *E2* locus is *GmGIa*, an ortholog of *GI* gene. The effect of the *E2* allele on flowering was relatively constant under different latitudinal locations [15]. The *e2* allele caused early flowering possibly through modulation of expression of *GmFT2a*, one of the soybean florigen genes [15,19]. Both *E3* and *E4* genes encode phytochrome A (PHYA) proteins. In *e3* allele, a large deletion of 13.33 kb occurs at the position after the third exon, leading to a nonfunctional phytochrome protein at the histidine kinase domain that has been confirmed to be important in signal transduction [14]. The *E3*, compared to the *E4* allele, is less sensitive to light quality as evidenced by similar flowering time phenotypes under long days with different light qualities [20,21]. However, the recessive *e3* allele is associated with the control of long-day insensitivity under fluorescent light with a high R:FR (red:far-red) ratio [5]. The recessive *e4* allele encodes a truncated GmphyA2 protein comprising 237 amino acids due to a 6238 bp insertion in exon 1 of *GmPHYA2* [13]. The *e4* allele requires the presence of *e3* to control long day-insensitivity under incandescent light with a low R:FR ratio [5,6].

Xia et al. (2012) successfully cloned the *E1* gene using a population derived from two Harosoy isolines carrying heterologous *E1* locus. The *E1* gene encodes a protein having a putative bipartite nuclear localization signal and a region distantly related to the B3 domain [16]. Allelic variation at each of four loci among 180 cultivars or accessions had a significant effect on flowering time as well as maturity time [22]. At least five recessive allelic variations (*e1-as*, *e1-nl*, *e1-fs*, *e1-re*, *e1-p*) have been identified at *E1* [16,23]. The *e1-nl* allele codes for a null mutation, in which about a 130 kb region including the entire *E1* gene (regulatory regions and transcribed region) has been deleted. The *E1* and *e1-as* alleles are two commonly found in modern cultivars in China, Japan and USA [22]. In the recessive *e1-as* allele, an early flowering phenotype might be ascribed to the loss of localization specificity of the E1 protein, which was resulted from a nonsynonymous substitution occurring in the putative nuclear localization signal [16]. The allele *e1-fs* has a 1-bp deletion in codon 17 leading to almost the entire B3 domain being truncated [16]. The mutations of *e1-re* and *e1-p* occur only at the 5’UTR region of the *E1* gene; and effects of both alleles on flowering time have not been well studied [23]. In this study, we identified a new *E1* allele *e1-b3a*, a 5 bp compound variation in the middle of the B3-like domain, and further tested the effects of this allele on flowering time using an F_2_ population.

Transcriptional abundance of the functional *E1* gene was significantly associated with flowering time. The flowering time phenotypes between different Harosoy *E1* near isogenic lines (NILs) were associated with the differential expression of the two *GmFT*-like genes both under SD and LD conditions, inferring that the *E1* locus suppresses flowering through the modulation of *GmFTs* expression [24]. A lower expression of the *E1* gene that was coupled with an elevated expression of *GmFT2a* or *GmFT5a* was observed in Kariyutaka, and in other Harosoy *E1* NILs with both loss-of-function alleles of *GmPHYA* (*e3* and *e4*) [16]. Similarly, in transgenic plants, a high expression of the *E1* gene resulted in suppressed expression of *GmFTs* [16]. Under SD conditions, the expression of the *E1* gene is highly suppressed. A bimodal diurnal expression pattern of the *E1* gene has been revealed under LD conditions [16]. But how the expression pattern is related to the photoperiodic length and the circadian clock remains unclear.

Although the genetic effects of the *E* genes on flowering time or maturity have been analyzed using the Harosoy and Clark NILs [10,11,15,21,22], the accuracy of this kind assessment may depend on the length of heterologous regions between NILs for *E1* and other *E* genes [24,25]. Since the molecular basis for four major *E* genes were unveiled, several research groups have analyzed the allelic variations of these genes among cultivars and accessions, and genetic effects of these variations on phenotypes have been tested [22,23,26]. About 62–66% variation in flowering time in 63 accessions could be explained by *E1* to *E4* [23]. However, the genetic effect of the *E1* gene on flowering time has not been confirmed directly in populations using functional markers generated from the *E1* to *E4* genes.

The reciprocal transfer experiment using the *E1* NILs suggested that the pre-inductive photoperiod-sensitive phase can be as early as 5–7 day post-planting [25]. In order to reveal some specific features or clues linking *E1* expression to photoperiodic length and circadian rhythm, we performed a diurnal expression analysis of the *E1* gene under constant light or dark after being transferred from LD or SD conditions on 16 days after emergence. We identified a new allele with variation in the middle of the B3-like domain of the *E1* gene, and further characterized the subcellular localization and functional effect of this allelic variation on flowering time in an F_2_ population. Also, the function of the *E1* gene and the interactions with other *E* genes were analyzed using F_2_ populations.

## Materials and Methods

### Plant materials

Harosoy-*E1* (L68-694, *E1e2E3E4e5*, PI547707) was originally from the US Department of Agriculture, Agricultural Research Service, National Plant Germplasm System; the cultivars Suzumaru (JP 67771, *e1-as*, *e2-ns*, *E3-Mi*, *E4*) and Kariyutaka (*E1*, *E2-in*, *e3-tr*, *e4-SORE-1*) were obtained from the Japanese National Institute of Agrobiological Sciences; Yanhuang 3 (*e1*-*b3a*, *E2-in*, *E3-Mi*, *E4*), Zhonghuang 39 (*E1*, *e2-ns*, *E3-Mi*, *E4*), and Moshidougong 503 (*e1-as*, *E2-in*, *e3-Mo*, *E4*) came from the National Key Facility for Crop Gene Resources and Genetic Improvement, Institute of Crop Sciences, Chinese Academy of Agricultural Sciences. Three F_2_ populations were derived from crosses of Kariyutaka (pollen) × Suzumaru (ovule), Kariyutaka (pollen) × Moshidougong 503 (ovule), and Yanhuang 3 (pollen) × Zhonghuang 39 (ovule). Successful crossings were confirmed using SSR markers.

### Diurnal expression pattern

For diurnal rhythmic expression analysis, we used an isogenic line Harosoy-*E1* [16]. Plants were grown in an artificial climate chamber under either SDs (12 h light: 12 h dark) or LDs (16 h light: 8 h dark) at 28°C under a light intensity of 200–300 μmol m^-2^ s^-1^. Plants were kept in SD or LD for 16 days (after emergence) before being transferred into continuous light (LL) or dark conditions (DD). Pieces of fully expanded trifoliate leaves from three different plants in each condition were sampled every 2 hours starting at dawn under SD, LD and LL conditions, and sampled every 4 hours under DD condition for real-time PCR analysis.

### Quantitative Real-time PCR

The total RNA was extracted using the TRIzol (Life Technologies) method. The isolated RNA was then subjected to reverse transcription using the TransScript II First-Strand cDNA Synthesis SuperMix (Transgene, Beijing, China) [22]. Quantitative real-time PCR was performed on each cDNA sample with the TransStart Top Green qPCR SuperMix (Transgene, Beijing, China) by Bio-Rad Chromo4 Detection System (Bio-Rad, USA) according to the manufacturer’s protocol [22]. The measured Ct values were converted to relative copy-numbers using the ΔΔCt method [27]. Amplification of *TUA5* (*Glyma05g29000*.*1*) was used as an internal control to normalize all data [22]. The RNA of 48h LL condition was included in each batch of quantitative real-time PCR for normalization.

### Genotyping of the *E* genes

A standard CTAB DNA extraction protocol was followed [28]. Except for *e1-b3a*, the genotyping of all parental cultivars or populations at the *E1*, *E2*, *E3* and *E4* loci was performed as described previously [24]. Electrophoresis was conducted by either agarose gel or high-efficiency genome scanning (HEGS) with non-denaturing 12% polyacrylamide separating gels and 5% stacking gels [29,30]. The gels were stained with GelStain (Transgene, Beijing, China) and visualized with the GelDoc XR Molecular Imager System (Bio-Rad, USA). For genotyping of *e1-b3a*, fragment (442 to 444 bp) was amplified using the TI primer pair (Forward: TCAGATGAAAGGGAGCAGTGTCAAAAGAAGT/ Reverse: TCCGATCTCATCACCTTTCC) [16]. After *Bfu*I digestion, the fragment generated from *e1-b3a* was cut into two fragments (334 and 108 bp) while the other genotypes (*E1*, *e1-as*, *e1-fs*) remained uncut.

### Subcellular localization

To obtain a C terminus fusion plasmid, we amplified a cDNA fragment containing the coding region without a stop codon by means of PCR using the primer pair of forward: CCATCGATAGATGAGCAACCCTTCAGATGAAAGG/reverse: GACTAGTCCACCTTTCCTGAGATCTC, from plasmids (pGEM-T Easy, Promega, USA) containing the *e1-b3a* sequence. With the aid of artificially introduced *Cla*I and *Spe*I sites, the amplicon was then inserted downstream of the CaMV 35S promoter and in-frame with the 5’ terminus of the *eGFP* gene into the pBSK derived vector [16]. The recombinant fusion plasmids were introduced into onion epidermal cells by means of particle bombardment as described previously [16] and were observed using an Olympus BX53 Fluorescence Microscope.

### Phenotypic observation

Phenotypic observations were conducted at three geographic locations: 1. Hailun: Hailun Research Station, Hailun County, Heilongjiang Province (47°26'N, 126°38'E); 2. Harbin: The Campus of the Northeast Institute of Geography and Agroecology, Harbin, Heilongjiang Province (45°70'N, 126°64'E); 3. Huaian: Huaiyin Institute of Agricultural Sciences of Xuhuai Region in Jiangsu, Huaian, Jiangsu Province (33°57'N, 119°04'E). The general environmental parameters including daylength and temperature for above three locations were previously described [22].

Flowering time of two populations derived from two crosses of Kariyutaka × Moshidougong 503 and Kariyutaka × Suzumaru was observed at Harbin in 2013 and 2014, and at Hailun in 2014. For the population of Yanhuang 3 × Zhonghuang 39, phenotypic observations were observed at Harbin in 2013 and 2014, and at Huaian in 2014.

In this study, the R1 stage refers the beginning of bloom when the opening of the first flower was found at any node on the main stem according to Fehr’s system [31]. Flowering time (R1) refers to the days from emergence to the R1 stage.

### Statistical Analysis

In order to statistically evaluate the effects of allelic variation *e1-b3a* and other alleles at the *E* loci on flowering time, maturity and other traits, genotypic and phenotypic data were analyzed using the programs SPSS [22]. The Type III Sum of Squares was used to test the effects between subjects.

## Result

### Diurnal expression patterns of the *E1* gene

**Fig 1:**
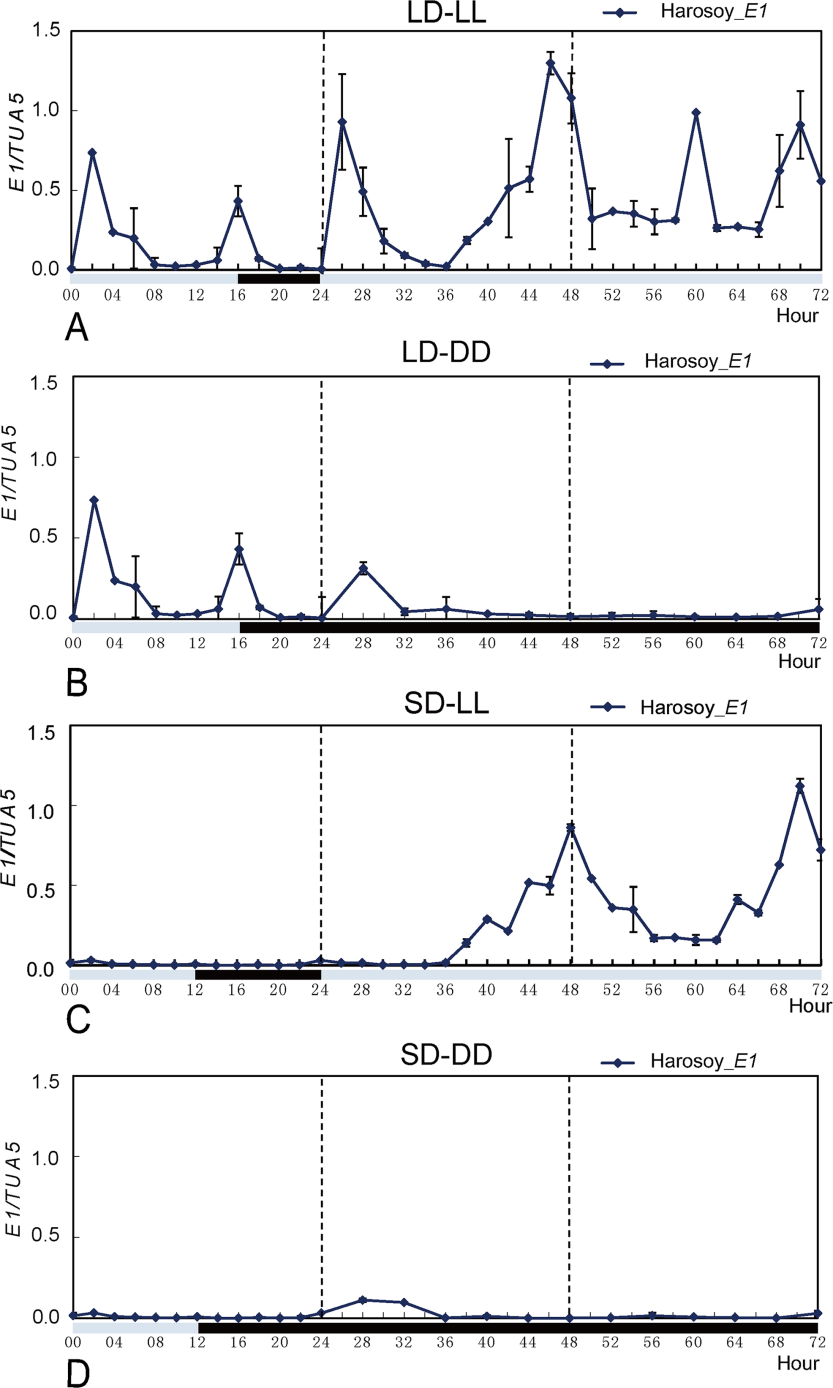
The expression patterns showing diurnal and circadian rhythm of the *E1* gene. Plants of Harosoy *E1* were grown under LDs (16 h light:8 h dark) or SDs (12 h light:12 h dark) before being shifted constant light (LL) or constant darkness (DD). A: LD-LL; B: SD-LL; C: LD-DD; D: SD-DD. Bars indicate standard error of the mean of three replicates.

Under the LD condition, the first peak of *E1* expression appeared around 2 hours after dawn (light was switched on), and the second peak occurred at 16 hours after dawn (Fig 1A). When plants were transferred from LD to continuous light (LL), the phase of the first peak was very much similar to that in LD, while the second peak appeared around 20 hours on the first subjective day, indicating that the second peak in LD might be gated by the starting of the dark phase at the 16th hour. On the second subjective day, the first peak appeared at the 12th hour, that is, an 8-hour phase lag was observed. Also the amplitude was changed where the basal expression level was elevated (Fig 1A).

On the first subjective day after plants were transferred from LD to continuous dark (DD), the first peak of the diurnal pattern appeared approximately at 4 hour, the phase became a little lagged with a lower magnitude. The second peak did not appear on the second subjective day, and the basal expression level was much lower (Fig 1B).

In SD, the basal expression level was lower with no notable peaks (Fig 1C). When plants were transferred from SD to LL, the elevated *E1* expression appeared about 12–14 hours after dawn, and peaked at 24 hours, and a second peak appeared around 22 hours on the second subjective day (Fig 1C). When plants were transferred from SD to DD, the expression pattern was similar to that in SD with no peak detected (Fig 1D).

The circadian expression of the *E1* gene showed a typical bimodal pattern in LD, with suppressed expression in SD. Continuous light elevated the basal expression level, while continuous darkness decreased expression of the *E1* gene.

### *e1-b3a* is a novel mutation of the *E1* gene

**Fig 2:**
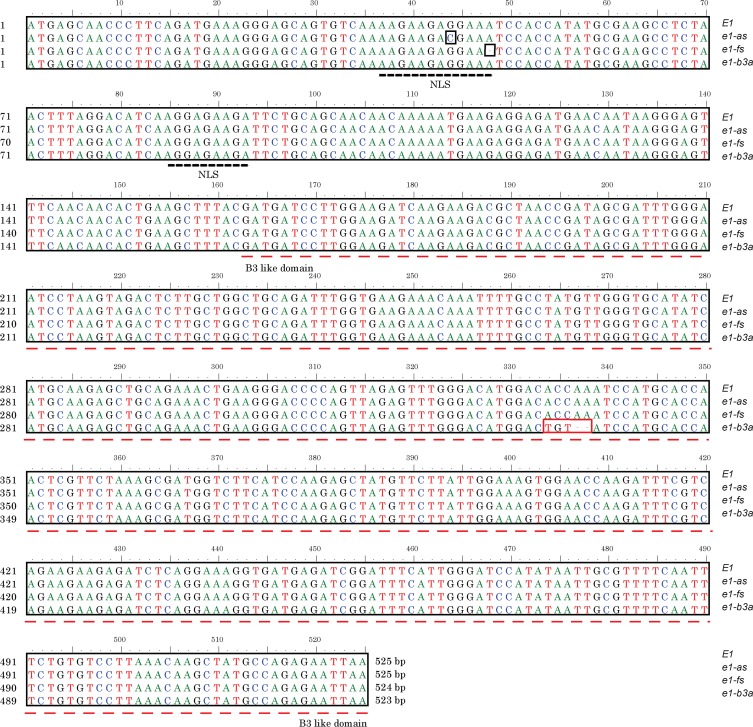
Alignment of *e1-ba3* with other known *E1* mutations. The SNP mutation site for *e1-as*, *e1-fs* are marked by black boxes; and the 5 bp mutation is marked with a red box. The bipartite nuclear localization sites are marked by black dashed line. The B3-like domain is also indicated with a dashed red line.

When we compared sequence of the *E1* gene amplified from different cultivars with the TI primer pair using HEGS, we identified a new 5bp (3 SNP and 2-bp deletion) mutation occurring in the middle of the B3-like domain of the *E1* gene in Yanhuang 3, a Chinese cultivar (Fig 2). This new mutation was referred to as *e1-b3a*. In comparison with the *E1* gene, the *e1-b3a* retains the intact bipartite NLS, but with only approximately half of the B3-like domain. The cultivar Yanhuang 3 with an early flowering and maturity time was bred in Yantai City (37°32'N, 121°23'E), Shandong Province. The duration of this cultivar (Yanhuang 3) from planting to harvest is about 90 days at Yantai City. Yanhuang 3 has a genotype of *E2-in*, *E3-Mi* and *E4* at the other characterized loci.

### Subcellular localization of e1-b3a

**Fig 3:**
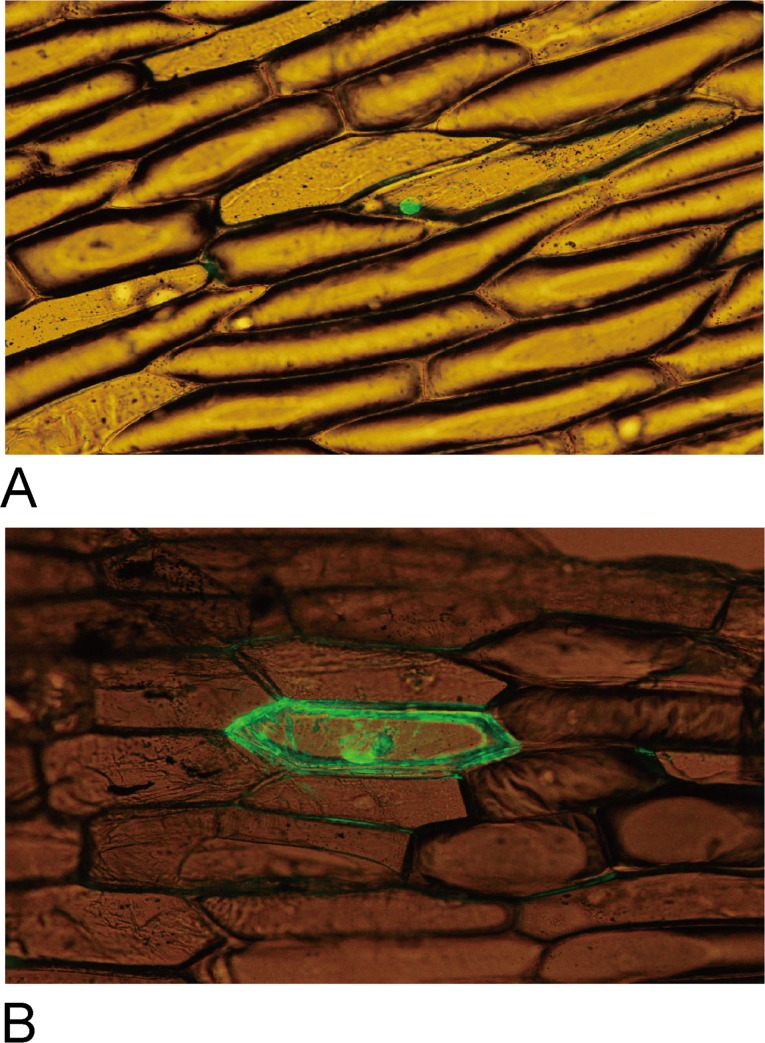
Subcellular distribution of the e1-b3a protein in onion epidermal cells. The e1-b3a-eGFP (A) and the control of eGFP alone (B) were produced transiently under the control of the CaMV 35S promoter and T-Nos in onion epidermal cells and were observed under a fluorescence microscope. The experiments were performed three times, a typical picture was presented.

In order to get some functional clue for the e1-b3a protein, we performed a subcellular localization experiment in the same way as previously described for the E1 and e1-as proteins [16]. The signal of the e1-b3a protein was also predominately located in the nucleus (Fig 3), indicating this mutation does not affect subcellular localization. The lost function of late flowering might result from the truncated B3-like domain due to the frameshift causing a premature stop codon at the middle of the B3-like domain.

### *e1-b3*a is an early flowering time mutation

**Fig 4:**
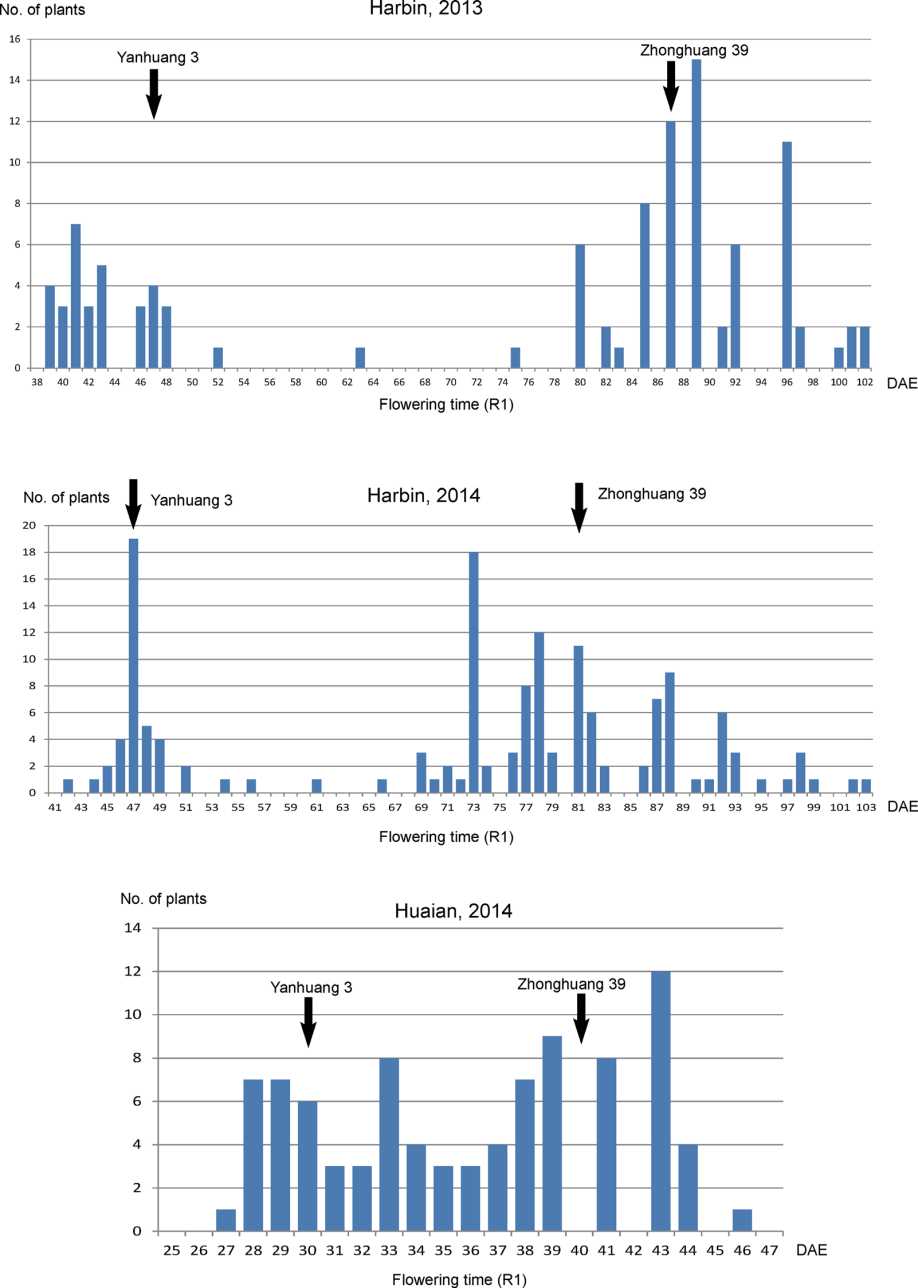
The distribution of flowering time (R1) of an F_2_ population derived from Yanhuang 3 × Zhonghuang 39. The flowering time (R1) for parents are indicated by arrows; DAE, days after emergence.

In the F_2_ population derived from Yanhuang 3 (*e1-b3a*, *E2-in*, *E3-Mi*, *E4*) × Zhonghuang 39 (*E1*, *e2-ns*, *E3-Mi*, *E4*), the parents are heterologous at both *E1* and *E2* loci, but with the same alleles at the *E3* and *E4* loci. The *E1* (*E1* vs *e1-b3a*) locus and *E2* (*E2-in* vs *e2-ns*) locus were significantly associated with flowering time (R1) (Fig 4) at both locations, Harbin (2013 and 2014) and Huaian (2014). The Cultivar Zhonghuang 39 flowered 81 to 86 days after emergence (DAE, sowed around May 1) in Harbin, and about 40 DAE (Sowed in June) in Huaian, while Yanhuang 3 flowered 47 DAE in Harbin and 30 DAE in Huaian (Fig 4).

On average, the *E1*/*E1*, *E1*/*e1-b3a* and *e1-b3a*/*e1-b3a* genotypes flowered 39.28 ± 1.38, 37.64 ± 0.61, and 29.22 ± 1.47 DAE, respectively (Table 1). *E1*/*E1* and *E1*/*e1-b3a* flowered 1.6 days apart, not significantly different showing dominance of *E1* over *e1-b3a*. In contract, the *e1-b3a/e1-b3a* genotype flowered significantly earlier than *E1*/*E1* and *E1*/*e1-b3a*. For the *E2* locus, the *E2/E2*, *E2/e2* and *e2/e2* genotypes flowered at 38.61 ± 0.75, 33.36 ± 0.80, and 31.44 ± 1.32 DAE, respectively. Meanwhile, in the northern location, Harbin in 2013, the *E1/E1*, *E1/e1-b3a* and *e1-b3a/e1-b3a* genotypes flowered on 92.75 ± 1.94, 76.05 ± 1.65, and 42.93 ± 2.06 DAE, respectively. The phenotypic difference in R1 between *E1* and *e1-b3a* was significantly larger in the northern site compared to the southern site (Table 1). For the *E2* locus, the *E2/E2*, *E2/e2* and *e2/e2* genotypes flowered at 79.42 ± 2.61, 72.32 ± 1.29, and 60.33 ± 2.61 DAE. A similar trend was observed at Harbin in 2014 (Table 1).

**Table 1:** The effect of *E1* and *E2* allelic variations on flowering time (R1) in an F2 population of Yanhuang 3 × Zhonghuang 39.

		Harbin, 2013		Harbin, 2014		Huaian, 2014	
		Mean	Std. Error	Mean	Std. Error	Mean	Std. Error
*E1*	*e2*	89.67	3.49	84.40	4.19	37.33	1.43
	*E2/e2*	89.33	2.47	83.86	2.00	37.10	1.11
	*E2*	96.00	2.37	93.63	3.32	43.40	1.57
*E1/e1-b3a*	*e2*	70.25	3.02	73.00	2.27	33.44	1.17
	*E2/e2*	83.94	2.01	75.59	1.47	37.23	0.69
	*E2*	91.00	2.47	81.90	2.10	42.25	1.24
*e1-b3a*	*e2*	41.40	3.82	47.75	3.32	28.00	3.50
	*E2/e2*	43.67	2.21	50.79	2.51	29.50	2.48
	*E2*	43.67	4.93	49.71	2.51	30.17	1.01

However, further two-way ANOVA analysis revealed that the *E1/E1*, *E1/e1-b3a* and *e1-b3a/e1-b3a* genotypes alleles performed differently depending on the genetic background of the *E2* alleles. *E1/E1 or E1/e1-b3a* suppressed flowering more efficiently in the *E2/E2* background compared to the *E2/e2 or e2/e2* background (Table 1). The interaction between *E1* and *E2*, however, did not reach a significant level (Table 2). At Harbin in 2013 and 2014, the interaction of *E1* and *E2* became significant at P<0.001 with the large effect of the *E1* locus (Table 2). This result indicates some allelic combinations at *E1* and *E2* loci in some environments have preferential effects on flowering time, although we have no clue at molecular level for the interaction between *E1* and *E2*.

**Table 2:** Statistical analysis of genetic effects of allelic variations at the *E1* and the *E2* loci on flowering time (R1) in an F_2_ population of Yanhuang 3 × Zhonghuang 39.

Location, Year	Factor	Type III Sum of Squares	df	Mean Square	F	Significance level
Harbin, 2013	Intercept	185644.17	1	185644.17	2543.21	0.000
	*E1*	23251.61	3	7750.54	106.18	0.000
	*E2*	2105.49	3	701.83	9.62	0.000
	*E1* × *E2*	3174.79	8	396.85	5.44	0.000
	Error	6423.64	88	73.00		
Harbin, 2014	Intercept	153654.47	1	153654.47	1747.19	0.000
	*E1*	25110.94	2	12555.47	142.77	0.000
	*E2*	1118.30	3	372.77	4.24	0.007
	*E1* × *E2*	1915.58	5	383.12	4.36	0.001
	Error	12312.13	140	87.94		
Huaian, 2014	Intercept	53739.83	1	53739.83	4375.76	0.000
	*E1*	682.22	3	227.41	18.52	0.000
	*E2*	489.73	3	163.25	13.29	0.000
	*E1* × *E2*	87.68	5	17.54	1.43	0.224
	Error	957.94	78	12.28		

### The effect of *E1* allelic variations on flowering time under different genetic backgrounds

**Fig 5:**
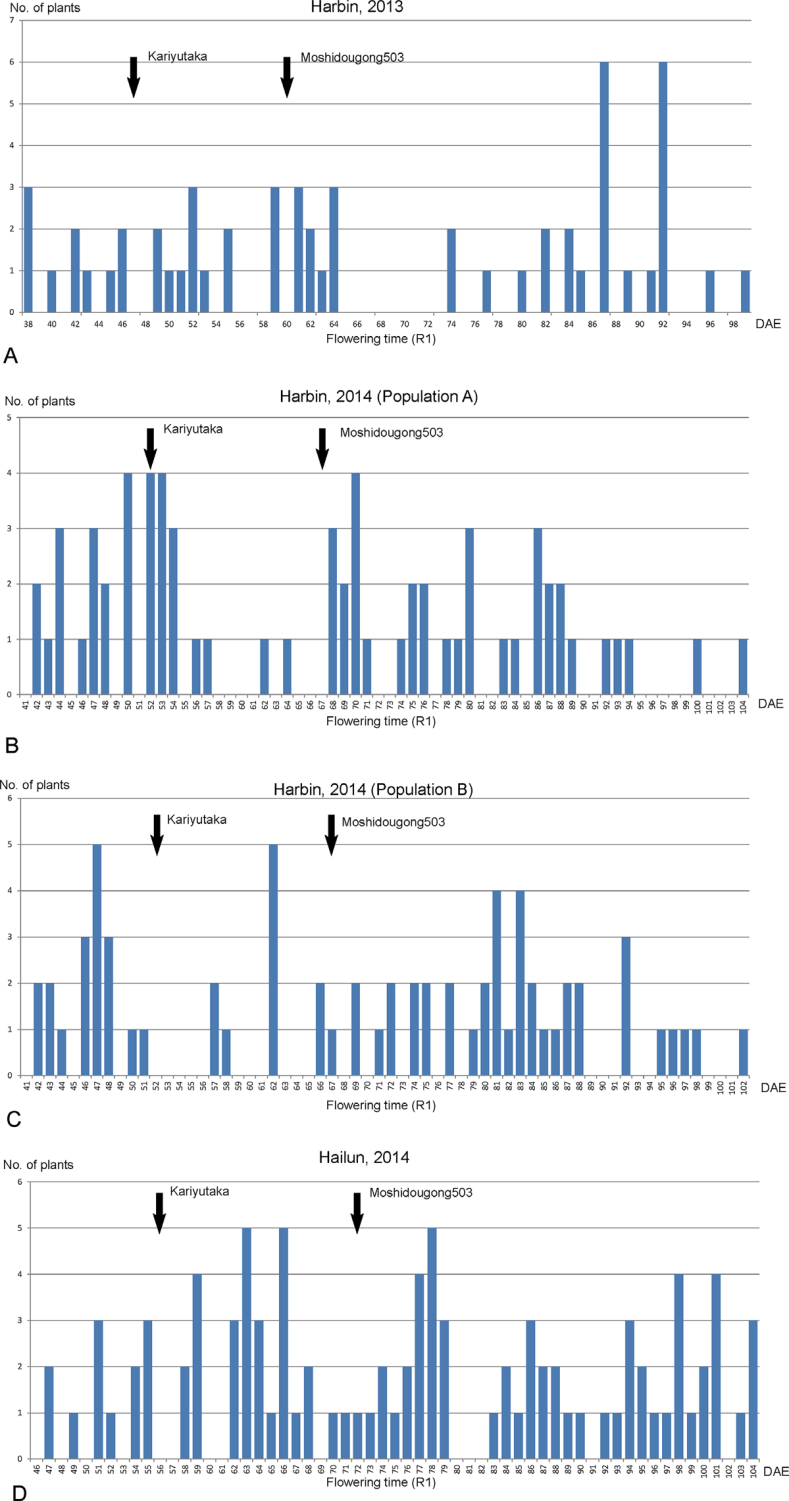
The distribution of flowering time (R1) of F_2_ populations derived from Kariyutaka × Moshidougong 503 at Harbin in 2013 (A), in 2014 (B and C), and at Hailun in 2014 (D). The flowering time (R1) for parents are indicated by arrows; DAE, days after emergence.

In the population Kariyutaka × Moshidougong 503, the parents are heterologous at the *E1*, *E3* and *E4* loci, but not at *E2* locus. The Cultivar Moshidougong 503 flowered about 72 DAE in Hailun, and 60 to 67 DAE in Harbin. Meanwhile, cultivar Kariyutaka flowered about 56 DAE in Hailun, and 47 to 52 DAE in Harbin (Fig 5). The impacts of *E1*, *E3* and *E4* alleles on flowering time reached statistical significance at both Harbin (in 2013 and 2014) and Hailun (in 2014).

The *E1* locus had a significant impact on R1 at Harbin (2013 and 2014) and Hailun (2014) at P<0.01 to P<0.001. The *E3* and *E4* loci also had significant impacts, though the magnitude was less than *E1*, on R1, with fluctuations between latitudinal sites and between years (S1 Table). The *E4* locus showed a rather larger impact on flowering time compared to the *E3* locus in this population since both *e3-Mo* and *e3-tr* alleles are recessive. The statistical significance at P value from 0.010 to 0.052 (S1 Table) might reflect the functional nuances between two recessive *E3* alleles.

**Fig 6:**
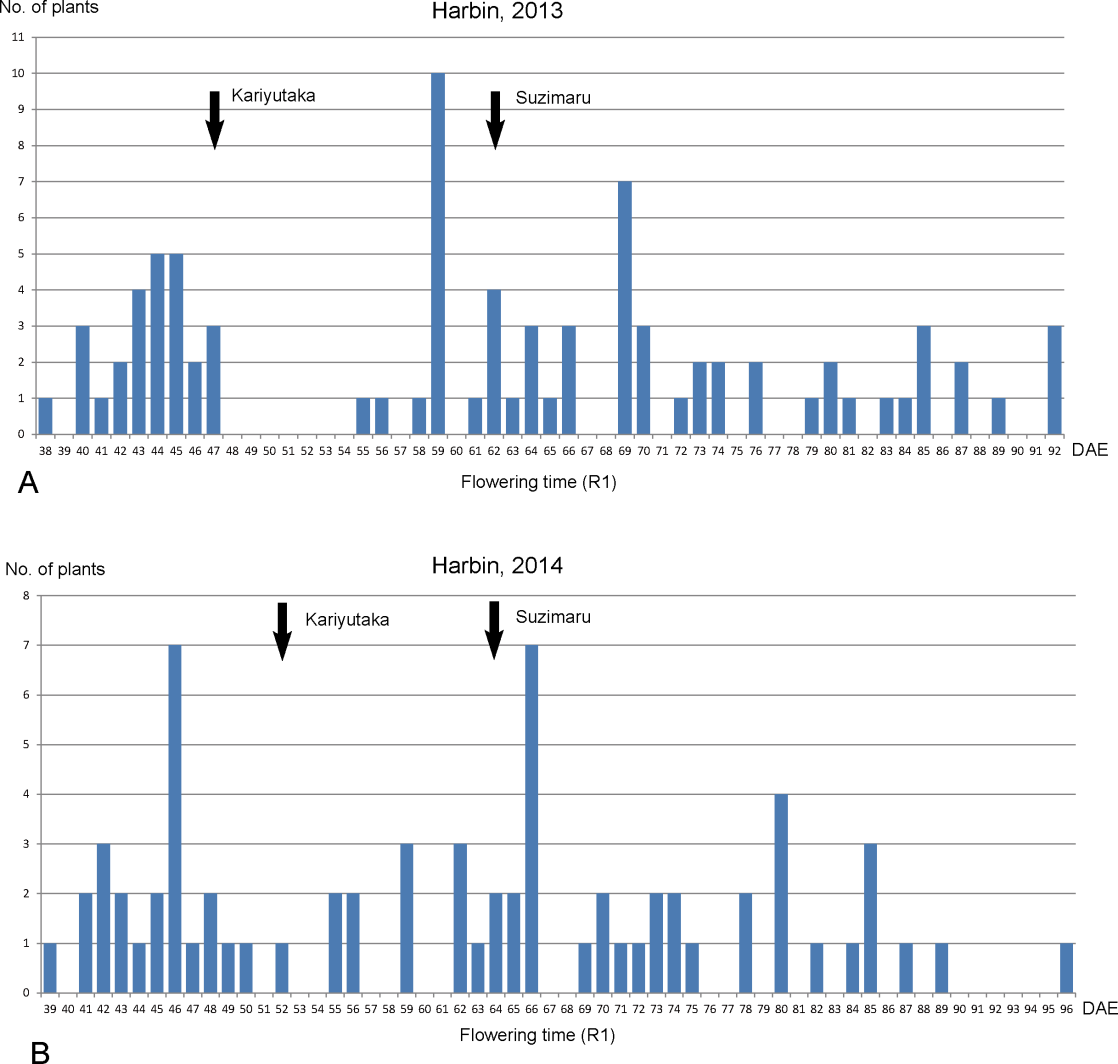
The distribution of flowering time (R1) of an F_2_ population derived from Kariyutaka × Suzumaru at Harbin in 2013 (A) and in 2014 (B). The flowering time (R1) for parents are indicated by arrows; DAE, days after emergence.

In the population of Kariyutaka × Suzumaru, both parents are heterologous at the *E1*, *E2*, *E3* and *E4* loci. Interestingly, strong transgressive segregation was detected in the F_2_ population although both parents showed relatively early flowering time phenotype. In Harbin in 2013 and 2014, cultivar Suzumaru flowered about 62 to 64 DAE, while Kariyutaka flowered 47 to 52 DAE (Fig 6).

All four loci have their influences on flowering time. Comparatively, the *E1* locus has the most significant impact on the flowering time (S2 Table).

## Discussion

### Basal expression level of the *E1* gene is associated with the photoperiodic length

Soybean cultivars flower early in SD, and differences between cultivars of different latitudinal origins become smaller or disappeared in SD. Generally, expression of the *E1* gene is suppressed in SD, and is significantly associated with flowering time among cultivars carrying the same *E1* or *e1-as* alleles [24]. In this study, we confirm the significant impact of the *E1* gene on flowering time and maturity. However, some photoperiodic sensibility is still remaining in cultivars carrying the *e1-nl* null allele, reflecting some other pathways mediating photoperiodic sensibility still exist when the *E1* gene is absent [26].

In this study, we revealed that the expression of the *E1* gene is promoted by long days. The diurnal expression pattern for *E1* in this study was similar to the typical bimodal expression described previously [16], except that the first peak appeared around 2 hours after dawn in this study compared to 4 hours in the previous study. The slight discrepancy for the first peak might be ascribed to the sampling intervals (2 hours in this study vs 4 hours in Xia et al.’s experiment). Judging from the rhythmic phasing and the magnitude of the expression patterns in LD-LL and LD-DD, the long night might be the key factor leading to suppressed *E1* expression. The rhythm could not be kept for more than one subjective day, indicating that the *E1* gene is somewhat, but not tightly, associated with the circadian clock.

### The *E1* gene underwent strong selection pressure

In this study, we identified a new type of mutation for the *E1* gene, which is suitable for studying *E1* function since only half of the B3-like domain remains in the mutant. The result reconfirmed that the B3-like domain is very important for the function of the *E1* gene. The mutation in the *e1-as* allele occurred at or near the bipartite NLS and the subcellular localization has been changed in comparison to *E1*. The mechanism leading to the lost function in the *e1-b3a* mutation is not the same as the *e1-as* mutation since *e1-b3a* is localized mainly in the nuclei. The *e1-as* allele is a typical leaky allele, keeping some partial function as a flowering suppressor. Xia et al. 2012 demonstrated the functional difference between *E1* and *e1-as* might result from subcellular localization as a putative transcription factor. In total, four types of mutations have been identified in the coding region of the *E1* gene, apart from the mutation identified from EMS generated library [16]. Additionally, some mutations in the promoter region might affect the expression level of the *E1* gene, thus leading to a changed phenotype of flowering time [23]. The various allelic variations, along with the expressional differences of the *E1* gene confer soybean cultivars a large flexibility to adapt to different latitudinal environments. Zhou et al. (2015) demonstrated that the *E1* gene underwent selection [32]. A strong signal at the *E1* locus was detected when comparing accessions from China with that from United States and Canada; and the distribution of mutant alleles was consistent with high latitude regions, including Korean, northern Japan, and northeastern China [32].

### Phenotypic performance of the *E1* gene is conditioned by genetic background and latitudinal location

Many studies have been conducted on the relationship between *E* loci using Harosoy and Clark NILs carrying heterologous *E* loci [21,22,24,33,34]. In this study, we used three biparental populations to mimic different genetic backgrounds for the *E2*, *E3* and *E4* loci. The allelic variations at the *E1* gene were significantly underlying flowering time in all three populations, and the magnitude of the impact was larger in northern latitudinal locations. The effects of the allelic variations at the *E2*, *E3* and *E4* loci generally reached statistical significances though with some fluctuations. The *E3* and *E4* loci differentially react with the light quality [21,35], which is consistent with the functional genes, GmPHYA3 and GmPHYA2, for the *E3* and *E4* loci. In the F_2_ population of Kariyutaka × Suzumaru, parent Kariyutaka flowered earlier than parent Suzumaru. As reported previously, the expression of the *E1* gene in Kariyutaka is suppressed possibly due to the genetic background of *e3* and *e4* [16]. A high segregation on flowering time phenotype is consistent with the occurrence of various genetic combinations at the four *E* loci. The magnitude of the genetic factors between different latitudinal locations may interact with many environmental factors, including photoperiodic length, temperature and light quality. Environmental changes e.g. global warming and air pollution, might also affect the performance of the *E* genes. Further studies on the functional mechanisms of the *E1* gene, other *E* genes, and new genes on controlling flowering time will enable us to understand more special and detailed features in photoperiodic flowering pathways in soybean.

## Supporting Information

S1 Table Statistical analysis of genetic effects of allelic variations at the *E1*, *E3*, and *E4* loci and their interactions on flowering time (R1) in an F_2_ population of Kariyutaka × Moshidougong 503. (DOCX)

S2 Table The statistic analysis of genetic effects at the *E1*, *E2*, *E3*, and *E4* loci and their interactions on flowering time (R1) in an F_2_ population of Kariyutaka × Suzumaru. (DOC)
