# Supplementary material for: Diurnal Expression Pattern, Allelic Variation, and Association Analysis Reveal Functional Features of the E1 Gene in Control of Photoperiodic Flowering in Soybean
Source: PLoS One. 2015 Aug 14;10(8):e0135909. doi: 10.1371/journal.pone.0135909 (PMC4537287; doi:10.1371/journal.pone.0135909)
Supplement: S1 Table — (DOCX) [file pone.0135909.s001.docx]

**S1 Table. Statistical analysis of genetic effects of allelic variations at the *E1*, *E3*, and *E4* loci and their interactions on flowering time (R1) in an F_2_ population of Kariyutaka × Moshidougong 503.**

| **Location(Year)** | **Factor** | **Type III Sum of Squares** | **df** | **Mean Square** | **F** | **Significance level** |
| --- | --- | --- | --- | --- | --- | --- |
| Harbin(2013) | Intercept | 100572.56 | 1 | 100572.56 | 941.37 | 0.000 |
|  | *E1* | 4155.32 | 3 | 1385.11 | 12.97 | 0.000 |
|  | *E3* | 929.71 | 3 | 309.90 | 2.90 | 0.052 |
|  | *E4* | 5110.65 | 3 | 1703.55 | 15.95 | 0.000 |
|  | *E1* × *E3* | 219.23 | 5 | 43.85 | 0.41 | 0.838 |
|  | *E1* × *E4* | 624.44 | 4 | 156.11 | 1.46 | 0.240 |
|  | *E3* × *E4* | 218.10 | 5 | 43.62 | 0.41 | 0.839 |
|  | *E1* × *E3* × *E4* | 140.07 | 3 | 46.69 | 0.44 | 0.728 |
|  | Error | 3098.25 | 29 | 106.84 |  |  |
| Harbin(2014a) | Intercept | 105746.35 | 1 | 105746.35 | 517.18 | 0.000 |
|  | *E1* | 3353.93 | 4 | 838.48 | 4.10 | 0.006 |
|  | *E3* | 2620.91 | 3 | 873.64 | 4.27 | 0.010 |
|  | *E4* | 891.16 | 3 | 297.05 | 1.45 | 0.240 |
|  | *E1* × *E3* | 945.78 | 4 | 236.44 | 1.16 | 0.342 |
|  | *E1* × *E4* | 2113.45 | 5 | 422.69 | 2.07 | 0.086 |
|  | *E3* × *E4* | 2919.92 | 4 | 729.98 | 3.57 | 0.013 |
|  | *E1* × *E3* × *E4* | 741.37 | 7 | 105.91 | 0.52 | 0.816 |
|  | Error | 9610.08 | 47 | 204.47 |  |  |
| Harbin(2014b) | Intercept | 115886.75 | 1 | 115886.75 | 1198.86 | 0.000 |
|  | *E1* | 4517.30 | 3 | 1505.77 | 15.58 | 0.000 |
|  | *E3* | 948.54 | 3 | 316.18 | 3.27 | 0.032 |
|  | *E4* | 2592.35 | 3 | 864.12 | 8.94 | 0.000 |
|  | *E1* × *E3* | 1485.23 | 4 | 371.31 | 3.84 | 0.010 |
|  | *E1* × *E4* | 933.59 | 5 | 186.72 | 1.93 | 0.112 |
|  | *E3* × *E4* | 126.52 | 4 | 31.63 | 0.33 | 0.858 |
|  | *E1* × *E3* × *E4* | 337.38 | 3 | 112.46 | 1.16 | 0.337 |
|  | Error | 3576.58 | 37 | 96.66 |  |  |
| Hailun(2014) | Intercept | 124249.55 | 1 | 124249.55 | 873.48 | 0.000 |
|  | *E1* | 5655.65 | 2 | 2827.83 | 19.88 | 0.000 |
|  | *E3* | 1331.08 | 2 | 665.54 | 4.68 | 0.052 |
|  | *E4* | 1406.42 | 3 | 468.81 | 3.30 | 0.000 |
|  | *E1* × *E3* | 322.59 | 4 | 80.65 | 0.57 | 0.838 |
|  | *E1* × *E4* | 1006.40 | 4 | 251.60 | 1.77 | 0.240 |
|  | *E3* × *E4* | 351.06 | 1 | 351.06 | 2.47 | 0.839 |
|  | *E1* × *E3* × *E4* | 5.25 | 2 | 2.63 | 0.02 | 0.728 |
|  | Error | 10668.51 | 75 | 142.25 |  |  |
