# Supplementary material for: Diurnal Expression Pattern, Allelic Variation, and Association Analysis Reveal Functional Features of the E1 Gene in Control of Photoperiodic Flowering in Soybean
Source: PLoS One. 2015 Aug 14;10(8):e0135909. doi: 10.1371/journal.pone.0135909 (PMC4537287; doi:10.1371/journal.pone.0135909)
Supplement: S2 Table — (DOC) [file pone.0135909.s002.doc]

**S2 Table. The statistic analysis of genetic effects at the *E1*, *E2*, *E3*, and *E4* loci and their interactions on flowering time (R1) in F_2_ population of Kariyutaka × Suzumaru.**

| **Location(Year)** | **Factor** | **Type III Sum of Squares** | **df** | **Mean Square** | **F** | **Significance level** |
| --- | --- | --- | --- | --- | --- | --- |
| Harbin(2013) | Intercept | 113316.76 | 1 | 113316.76 | 1576.54 | 0.000 |
|  | *E1* | 1300.50 | 2 | 650.25 | 9.05 | 0.001 |
|  | *E2* | 1261.30 | 4 | 315.37 | 4.39 | 0.008 |
|  | *E3* | 74.87 | 3 | 24.96 | 0.350 | 0.791 |
|  | *E4* | 161.74 | 3 | 53.92 | 0.75 | 0.533 |
|  | *E1* × *E2* | 60.21 | 1 | 60.21 | 0.84 | 0.369 |
|  | *E1* × *E3* | 19.20 | 1 | 19.20 | 0.27 | 0.610 |
|  | *E1* × *E4* | 266.41 | 2 | 133.21 | 1.85 | 0.178 |
|  | *E2* × *E3* | 90.75 | 1 | 90.75 | 1.26 | 0.272 |
|  | *E2* × *E4* | 500.08 | 4 | 125.01 | 1.74 | 0.174 |
|  | *E3* × *E4* | 188.67 | 2 | 94.33 | 1.31 | 0.288 |
|  | Error | 1725.04 | 24 | 71.88 |  |  |
| Harbin(2014) | Intercept | 48416.35 | 1 | 48416.35 | 666.94 | 0.000 |
|  | *E1* | 993.70 | 2 | 496.85 | 6.84 | 0.023 |
|  | *E2* | 728.50 | 3 | 242.83 | 3.35 | 0.085 |
|  | *E3* | 1099.80 | 2 | 549.90 | 7.58 | 0.018 |
|  | *E4* | 595.08 | 3 | 198.36 | 2.73 | 0.123 |
|  | *E1* × *E2* | 270.57 | 3 | 90.19 | 1.24 | 0.364 |
|  | *E1* × *E3* | 1.14 | 1 | 1.14 | 0.02 | 0.904 |
|  | *E1* × *E4* | 14.00 | 1 | 14.00 | 0.19 | 0.674 |
|  | *E2* × *E3* | 187.14 | 2 | 93.57 | 1.29 | 0.334 |
|  | *E2* × *E4* | 1.79 | 1 | 1.79 | 0.03 | 0.880 |
|  | *E3* × *E4* | 92.50 | 2 | 46.25 | 0.64 | 0.557 |
|  | Error | 508.17 | 7 | 72.60 |  |  |
